# Supplementary material for: Multi‐dataset identification of innovative feature genes and molecular mechanisms in keratoconus
Source: J Cell Mol Med. 2024 Sep 19;28(18):e70079. doi: 10.1111/jcmm.70079 (PMC11412914; doi:10.1111/jcmm.70079)
Supplement: Supplementary file 2 — Material S2. [file JCMM-28-e70079-s002.zip › 7.GSEA analysis/3.DBNDD1/KEGG_ENDOMETRIAL_CANCER.html]

Details for gene set KEGG\_ENDOMETRIAL\_CANCER[GSEA]

|  || Dataset | DBNDD1.group.cls#h\_versus\_l |
| Phenotype | group.cls#h\_versus\_l |
| Upregulated in class | h |
| GeneSet | KEGG\_ENDOMETRIAL\_CANCER |
| Enrichment Score (ES) | 0.17741023 |
| Normalized Enrichment Score (NES) | 0.71387726 |
| Nominal p-value | 0.81930184 |
| FDR q-value | 1.0 |
| FWER p-Value | 1.0 |
Table: GSEA Results Summary

  

Fig 1: Enrichment plot: KEGG\_ENDOMETRIAL\_CANCER      
 Profile of the Running ES Score & Positions of GeneSet Members on the Rank Ordered List

  

| SYMBOL | TITLE | RANK IN GENE LIST | RANK METRIC SCORE | RUNNING ES | CORE ENRICHMENT || 1 | PIK3R5 | na | 35 | 0.708 | 0.1774 | Yes |
| 2 | ELK1 | na | 3923 | 0.216 | 0.1197 | No |
| 3 | PIK3R1 | na | 6912 | 0.126 | 0.0654 | No |
| 4 | SOS1 | na | 8586 | 0.093 | 0.0405 | No |
| 5 | AKT2 | na | 9388 | 0.080 | 0.0376 | No |
| 6 | CCND1 | na | 9676 | 0.076 | 0.0484 | No |
| 7 | MAPK3 | na | 9865 | 0.073 | 0.0615 | No |
| 8 | MAP2K2 | na | 10931 | 0.059 | 0.0456 | No |
| 9 | PTEN | na | 10959 | 0.059 | 0.0596 | No |
| 10 | PDPK1 | na | 11670 | 0.050 | 0.0518 | No |
| 11 | PIK3R2 | na | 11699 | 0.050 | 0.0636 | No |
| 12 | CTNNA2 | na | 11866 | 0.048 | 0.0710 | No |
| 13 | ERBB2 | na | 13132 | 0.037 | 0.0439 | No |
| 14 | SOS2 | na | 13580 | 0.033 | 0.0395 | No |
| 15 | TP53 | na | 13962 | 0.030 | 0.0361 | No |
| 16 | KRAS | na | 14127 | 0.029 | 0.0387 | No |
| 17 | PIK3R3 | na | 14144 | 0.029 | 0.0455 | No |
| 18 | BRAF | na | 15471 | 0.019 | 0.0122 | No |
| 19 | CTNNB1 | na | 16378 | 0.013 | -0.0106 | No |
| 20 | PIK3CA | na | 17073 | 0.009 | -0.0284 | No |
| 21 | CTNNA1 | na | 18276 | 0.001 | -0.0628 | No |
| 22 | CDH1 | na | 18305 | 0.001 | -0.0635 | No |
| 23 | PIK3CB | na | 18338 | 0.000 | -0.0643 | No |
| 24 | HRAS | na | 18984 | -0.004 | -0.0818 | No |
| 25 | RAF1 | na | 19005 | -0.005 | -0.0812 | No |
| 26 | AKT1 | na | 19066 | -0.005 | -0.0817 | No |
| 27 | EGF | na | 19333 | -0.007 | -0.0875 | No |
| 28 | AXIN1 | na | 19524 | -0.009 | -0.0908 | No |
| 29 | CASP9 | na | 19881 | -0.012 | -0.0981 | No |
| 30 | TCF7L2 | na | 20073 | -0.014 | -0.1001 | No |
| 31 | BAD | na | 20453 | -0.017 | -0.1068 | No |
| 32 | APC2 | na | 21518 | -0.027 | -0.1306 | No |
| 33 | EGFR | na | 22041 | -0.033 | -0.1375 | No |
| 34 | GRB2 | na | 22294 | -0.035 | -0.1359 | No |
| 35 | GSK3B | na | 22452 | -0.037 | -0.1312 | No |
| 36 | MYC | na | 22660 | -0.039 | -0.1273 | No |
| 37 | MAPK1 | na | 22705 | -0.040 | -0.1186 | No |
| 38 | ILK | na | 22715 | -0.040 | -0.1088 | No |
| 39 | MLH1 | na | 23263 | -0.046 | -0.1129 | No |
| 40 | FOXO3 | na | 23455 | -0.049 | -0.1061 | No |
| 41 | TCF7 | na | 23571 | -0.051 | -0.0966 | No |
| 42 | APC | na | 24380 | -0.062 | -0.1044 | No |
| 43 | ARAF | na | 24817 | -0.068 | -0.0998 | No |
| 44 | PIK3CD | na | 24971 | -0.070 | -0.0865 | No |
| 45 | TCF7L1 | na | 26813 | -0.104 | -0.1135 | No |
| 46 | NRAS | na | 27014 | -0.108 | -0.0920 | No |
| 47 | CTNNA3 | na | 27076 | -0.110 | -0.0661 | No |
| 48 | PIK3CG | na | 27455 | -0.118 | -0.0473 | No |
| 49 | AXIN2 | na | 28854 | -0.153 | -0.0491 | No |
| 50 | MAP2K1 | na | 29246 | -0.164 | -0.0190 | No |
| 51 | LEF1 | na | 32857 | -0.317 | -0.0430 | No |
| 52 | AKT3 | na | 33656 | -0.385 | 0.0309 | No |
Table: GSEA details [plain text format]

  

Fig 2: KEGG\_ENDOMETRIAL\_CANCER      
 Blue-Pink O' Gram in the Space of the Analyzed GeneSet

  

Fig 3: KEGG\_ENDOMETRIAL\_CANCER: Random ES distribution      
 Gene set null distribution of ES for **KEGG\_ENDOMETRIAL\_CANCER**
